# Mathematical Modeling Reveals That Sucrose Regulates Leaf Senescence via Dynamic Sugar Signaling Pathways

**DOI:** 10.3390/ijms23126498

**Published:** 2022-06-10

**Authors:** Muhammad Asim, Quaid Hussain, Xiaolin Wang, Yanguo Sun, Haiwei Liu, Rayyan Khan, Shasha Du, Yi Shi, Yan Zhang

**Affiliations:** 1 Key Laboratory of Tobacco Biology and Processing, Ministry of Agriculture and Rural Affairs, Tobacco Research Institute, Chinese Academy of Agricultural Sciences, Qingdao 266101, China; asim.ktk91@aup.edu.pk (M.A.); wangxiaolin@caas.cn (X.W.); sunyanguo@caas.cn (Y.S.); liuhaiwei@caas.cn (H.L.); rayyanswb@gmail.com (R.K.); wdsh0405@163.com (S.D.); 2 State Key Laboratory of Subtropical Silviculture, Zhejiang A&F University, 666 Wusu Street, Hangzhou 311300, China; quaid_hussain@yahoo.com; 3 Graduate School of Chinese Academy of Agricultural Science, Beijing 100081, China

**Keywords:** sucrose concentration, sugar signaling dynamics, glucose, trehalose-6-phosphate, *SnRK1*, senescence

## Abstract

Sucrose (Suc) accumulation is one of the key indicators of leaf senescence onset, but little is known about its regulatory role. Here, we found that application of high (120–150 mM) and low levels (60 mM) of Suc to young leaf (YL) and fully expanded leaf (FEL) discs, respectively, decreased chlorophyll content and maximum photosynthetic efficiency. Electrolyte leakage and malondialdehyde levels increased at high Suc concentrations (90–120 mM in YL and 60 and 150 mM in FEL discs). In FEL discs, the senescence-associated gene *NtSAG12* showed a gradual increase in expression with increased Suc application; in contrast, in YL discs, *NtSAG12* was upregulated with low Suc treatment (60 mM) but downregulated at higher levels of Suc. In YL discs, trehalose-6-phosphate (T6P) accumulated at a low half-maximal effective concentration (EC50) of Suc (1.765 mM). However, T6P levels declined as trehalose 6 phosphate synthase (TPS) content decreased, resulting in the maximum velocity of sucrose non-fermenting-1-related protein kinase (SnRK) and hexokinase (HXK) occurring at higher level of Suc. We therefore speculated that senescence was induced by hexose accumulation. In FEL discs, the EC50 of T6P occurred at a low concentration of Suc (0.9488 mM); T6P levels progressively increased with higher TPS content, which inhibited SnRK activity with a dissociation constant (*K_d_*) of 0.001475 U/g. This confirmed that the T6P–SnRK complex induced senescence in detached FEL discs.

## 1. Introduction

Senescence is the last stage of plant leaf growth and development [1], and is affected by the complex interplay between external and internal factors. These factors include exogenous environmental signals, endogenous phytohormone contents, and leaf age [2,3]. Senescence is a programmed process that affects material recovery and recycling. Sugars are substrates for carbon mobilization and reallocation [4]. The cellular pathways involved in transport, metabolism, and allocation of assimilates and sugars, primarily sucrose (Suc) and hexoses, are closely connected to regulation of carbon partitioning at the whole-plant level [5]. Plant growth and development are tightly regulated in response to environmental factors that affect photosynthetic carbon availability in the form of Suc. Suc transduction and translocation have significant regulatory functions during leaf senescence, reinforcing the hypothesis that leaf senescence is induced or enhanced by sugar deficiency [6]. However, other data support the opposing theory, that leaf senescence is caused or improved by rising sugar levels [7]. For example, sugar levels are higher in senescing tobacco leaves than in younger or mature but nonsenescing tobacco leaves [8]. Suc tends to be metabolized quickly, and it is therefore unclear if plant responses are due to the presence of Suc itself or to products derived from Suc breakdown (e.g., glucose (Glc), glucose 6 phosphate (G6P), and trehalose 6 phosphate (T6P)) [9,10].

Suc was first hypothesized to be a plant signaling molecule several decades ago [11]. Suc signaling has since been examined in the context of metabolic activities, and considerable evidence shows that Suc signals may govern a wide range of developmental events throughout the plant life cycle [11]. Many developmental processes such as flowering, circadian clock regulation, and senescence depend on Suc signaling [9]. During plant growth, the young, immature leaves act as significant sugar sinks, whereas mature and senescing leaves act as metabolite sources [12]. Plant developmental transition requires ample carbon, which can be sensed through sugar signaling pathways [13]. Hexokinase-1 (*HXK1)* responds to high carbon levels, whereas T6P, Snf1-related protein kinase-1 (*SnRK1*), the C/S1 bZIP transcription factor, and the target of rapamycin (TOR) are involved in sensing sugar starvation [13]. Suc is broken down into fructose and Glc by neutral invertase, which is abundantly expressed in leaf vasculature and has various functions in plant growth [14]. T6P is the precursor of the trehalose biosynthetic pathway and a key signaling molecule that governs plant growth and development in response to carbon availability [15]. The T6P signaling pathway is essential in leaf senescence. For instance, decreased levels of T6P in the *osmoregulatory trehalose synthesis* (otsB) gene led to late senescence phenotypes [16]. Suc levels affect the accumulation of T6P, thus determining the plant sugar status. T6P levels are 15-fold higher in older leaves compared to younger leaves [16]. *SnRK1* is a key factor in coordinating sugar and hormone signals; it is central to regulation of cellular responses to endogenous energy and the carbon state [17,18]. *SnRK1* activity is affected by T6P in plants [16,19]. Other sugars, such as G6P and glucose 1-phosphate (G1P), inhibit *SnRK1* activity in young tissues through the action of an unrelated protein [20]. Overexpression of the *SnRK1* gene *KIN10* leads to delayed senescence [16]; *SnRK1* and the transcription factor bZIP-11 have a synergistic effect in regulating the expression of senescence-related genes [21]. Hexose accumulation in plants is sensed by *HXK1* [22], which catalyses hexose phosphorylation and accelerates leaf senescence [23]. In *Arabidopsis*, the catalytically inactive *HXK1* mutants *S177A* and *G104D* suggested that HXK1 participates in signaling of Glc availability, which is independent of its function in Glc metabolism [24]. HXK is well-known for involvement in feedback regulation of sugar accumulation [25]. In addition, HXK catalyses the crucial processes of Glc and fructose phosphorylation, and has been shown to trigger senescence [24].

Mathematical modeling has emerged in recent years as a useful technique to understand and anticipate the mechanistic behavior of a biological system using equations that represent the underlying biochemical, biophysical, and mathematical features [26,27]. The Michaelis–Menten equation has been widely used over several decades to estimate the kinetic parameters of an enzyme from the reaction progress curve of a specific substrate [28]. The Hill equation is a rate law that is used to simulate sigmoidal responses in biological interactions; the equation describes biomolecular interactions in which two binding molecules cooperate [29]. The half-maximal effective concentration (EC50) is a ligand response indicator that is equal to the molar concentration of an agonist or substrate that produces 50% of the maximum possible effect [30]. For example, the predicted affinity of the G-protein coupled receptor Gpr1 is roughly 40 times higher for Suc than for Glc; the EC50 for Suc is only about 0.5 mM, whereas the EC50 for Glc is ~20 mM [31,32]. Bazzone et al. (2021) observed sugar binding and transport in real time to estimate the Michaelis constant (*K_m_*), maximum uptake rate (*V_max_*), and dissociation constant (*K_d_*) values for various sugar substrates such as Glc, Suc, mannose, and fructose [33]. *K_d_* could be determined for these substrates due to a conserved sugar-induced electrogenic conformational change inside membrane transporters of the major facilitator superfamily [33]. Another study revealed that the concentrations of neutral sugars (such as Glc, galactose, and arabinose) that yield half-maximal induction of *vir* gene expression are several orders of magnitude higher than expected based on the *K_d_* for binding to the chromosomal virulence gene E (*ChvE*) [34]. In apples and tomatoes, MdERDL6-1-mediated glucose efflux to the cytosol enhanced sugar buildup in the vacuole with a *K_m_* value of 21.7 mM and a *V_max_* of 1.214 mmol⋅h^−1^⋅mL^−1^ [35]. Enzyme kinetics equations were therefore used in this study to understand the reactions involving Suc transition in detached young leaf (YL) and fully expanded (FEL) discs. 

Tobacco was selected for this study because it has long been used as a model plant for biological research and has a chronologically ordered senescence mechanism in the leaves. It was previously posited that Suc lacks a signaling role because it is metabolized very quickly [10]. This quick metabolism means that it has been unclear whether plant responses are caused by Suc itself or by Suc degradation byproducts (e.g., Glc, G6P, and T6P). Here, enzyme kinetic equations were used to investigate dose-dependent reactions involving exogenous Suc, sugar precursors, and the related signaling dynamics that regulate leaf senescence. The roles of HXK signaling and SnRK metabolic regulation were studied in detached tobacco YL and FEL discs.

## 2. Results

### 2.1. Exogenous Suc Affects Growth and Stimulates Senescence in Detached Leaf Discs 

**Figure 1:**
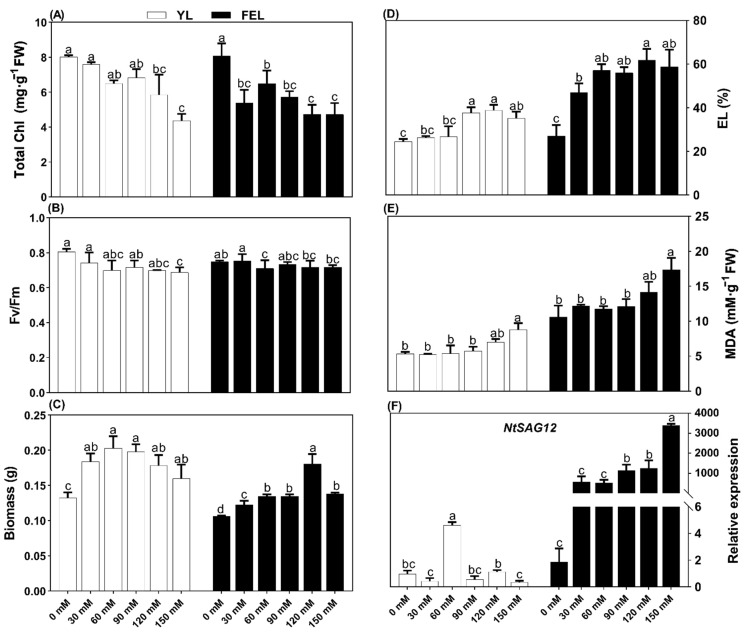
Characteristics of detached *N. tabacum* leaf discs in liquid culture with added sucrose (Suc): (**A**) total chlorophyll (Chl) content; (**B**) maximum photosynthetic efficiency (F_v_/F_m_); (**C**) biomass; (**D**) percent electrolyte leakage (EL); (**E**) malondialdehyde (MDA); (**F**) relative expression of *NtSAG12*. Lowercase letters above each bar represent statistically significant differences between treatments groups at *p* ≤ 0.05. Error bars indicate standard deviation of three replicates.

Chlorophyll (Chl) content gradually decreased in both YL and FEL discs as the concentration of Suc increased. Compared to untreated discs, YL samples treated with 120 and 150 mM Suc showed 27.0% and 45.5% decreases in Chl content, respectively. In FEL discs treated with 30 and 150 mM Suc, Chl content decreased by 19.4% and 41.2%, respectively, compared to untreated samples (Figure 1A). YL and FEL discs gradually turned yellow in response to Suc treatment. Total Chl content was more responsive to Suc in FEL than in YL discs. There was a minor decrease in maximum photosynthetic efficiency (F_v_/F_m_) in YL and virtually no change in FEL discs. In YL discs, those treated with 150 mM Suc showed a reduction in F_v_/F_m_ of 14.7% compared to untreated samples. FEL discs treated with 60 mM Suc showed a decrease in F_v_/F_m_ of 5.2% compared to untreated samples. In summary, a low Suc concentration (60 mM) affected F_v_/F_m_ in the FEL discs, whereas a high Suc concentration (150 mM) affected F_v_/F_m_ in the YL discs (Figure 1B). 

The effects of exogenous Suc on biomass accumulation were also analyzed. Suc application significantly increased both YL and FEL biomass; in YL discs, treatment with 30, 60, 90, 120, and 150 mM Suc increased biomass accumulation by 38.7, 53.2, 34.7, 49.4, and 20.5%, respectively. Biomass was consistently lower in FEL discs, but increased by 15.4, 26.7, 26.7, 70.2, and 30.0% in response to 30, 60, 90, 120, and 150 mM Suc, respectively (Figure 1C). 

### 2.2. Effects of Suc Concentration on Membrane Permeability of Detached Leaves

Massive fluctuations in electrolytic leakage (EL) and malondialdehyde (MDA) content are common features of leaf senescence in plants [36]. To investigate Suc-induced leaf senescence, EL was studied in detached leaf discs treated with various concentrations of Suc. It was found that Suc treatment significantly affected EL induction in the detached leaf discs. In the YL discs, EL significantly increased by 57.1 and 58.4% at 90 and 120 mM Suc, respectively. Similarly, in FEL discs, EL increased by 110.2 and 127.6% at 30 and 120 mM Suc, respectively, compared to untreated discs (Figure 1D). MDA levels were also significantly increased in response to 150 mM Suc treatment in both YL and FEL discs (by 8.8 and 17.3%, respectively) (Figure 1E). The results indicated that exogenous Suc, especially at 150 mM, increased lipid peroxidation in YL and FEL discs. EL data also indicated that low Suc levels affected membrane permeability in both YL and FEL discs, clarifying that exogenous Suc uptake affects cell permeability during senescence. 

### 2.3. Effects of Exogenous Suc on Expression of the Senescence-Associated Gene NtSAG12 

Expression of the senescence-associated gene *NtSAG12* was next studied in detached *N. tabacum* YL and FEL discs. In YL samples, *NtSAG12* expression was not significantly altered by Suc application; the exception was treatment with 60 mM Suc, which led to a 3.7-fold higher expression of *NtSAG12*. In contrast, FEL discs showed significant upregulation of *NtSAG12* with increased Suc levels: 609.4-, 669.6-, and 1821.7-fold higher expression at 90, 120, and 150 mM Suc, respectively. These results revealed an inconsistent response of *NtSAG12* to Suc in YL discs, with an initial increase in expression followed by a decrease. In contrast, FEL consistently responded to *NtSAG12* induction with changes in biochemical and physiological traits that could induce senescence in the detached *N. tabacum* leaf discs. 

### 2.4. Effect of Suc on Regulation of Sugar Metabolic Pathways during Senescence 

**Figure 2:**
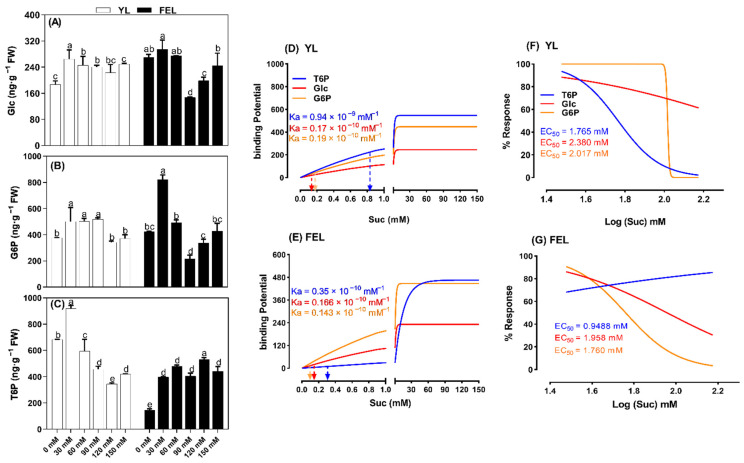
Evaluation of glucose (Glc) (**A**), glucose 6-phosphate (G6P) (**B**), and trehalose 6-phosphate (T6P) (**C**) content in detached *N. tabacum* young leaf (YL) and fully emerged leaf (FEL) discs in response to sucrose (Suc) treatment. Lowercase letters indicate statistically significant differences (*p* < 0.05) between treatment groups. The association constant (*K_a_*) values (**D**,**E**) and dose-dependent Suc response curves (**F**,**G**) for Glc, G6P, and T6P are represented by red, gold, and blue lines, respectively.

To determine the Suc uptake potential of detached tobacco YL and FEL discs, the metabolic conversion of exogenous Suc into Glc, G6P, and T6P was studied. The results indicated that in the YL discs, Glc content increased by 41.6, 31.4, and 28.7% at 30, 60, and 90 mM Suc treatments, respectively, compared to untreated discs. In the FEL discs, Glc content increased by 9.3% at 30 mM Suc, but sharply decreased at 60 and 90 mM before slightly increasing again at 120 and 150 mM Suc (Figure 2A). These results showed the differing effects of Suc supply on Suc uptake and Glc accumulation in detached leaf discs. YL discs turned yellow in response to increasing Suc concentrations, thus affecting the growth transition and maturation. However, FEL discs proceeded directly to senescence when the sugar supply was low and internal sugar levels decreased. 

Glc is broken down into G6P and T6P shortly after synthesis. Our results showed that in YL discs, Suc treatment caused an initial increase in G6P content (of 33.1, 33.4, and 37.9% at 30, 60, and 90 mM Suc, respectively). However, this was followed by a sharp decrease at 120 and 150 mM Suc. In FEL discs, the G6P content first increased at a low concentration of Suc (by 94.4% at 30 mM), but progressively decreased with increased Suc levels (e.g., −48.8% at 90 mM) (Figure 2B). 

The Suc–T6P nexus concept postulates that T6P is both a signal and a negative-feedback regulator of Suc levels in plants, helping to maintain Suc levels within an optimal range [37]. Our results showed that in the detached YL discs, T6P content progressively decreased in response to increasing Suc supply (−13.2, −33.2, −49.4, and −38.6% at 60, 90, 120, and 150 mM Suc, respectively, compared to untreated discs) (Figure 2C). The exception was the 30 mM Suc treatment, with which the T6P content significantly increased by 34.6%. Suc affected T6P accumulation differently in detached FEL discs compared to YL discs; T6P increased in response to Suc in FEL discs. It is noteworthy that the maximum T6P value occurred at 120 mM Suc and represented a 262.0% increase compared to untreated samples. These results showed that the Suc uptake potential differed between detached YL and FEL discs. In the YL discs, 120 mM Suc treatment severely hindered T6P accumulation, whereas 120 mM Suc induced T6P accumulation in detached FEL discs. 

To understand the differing responses of T6P signaling to Suc levels, we performed mathematical kinetic analyses, which have become popular tools for evaluating networks with outputs that are difficult to grasp intuitively [38]. We found that Glc and phosphate derivatives had different EC50 values in samples treated with different concentrations of Suc. In YL discs, the EC50 value of T6P occurred at a relatively low concentration of Suc (1.765 mM) compared to Glc and G6P (2.380 and 2.017 mM Suc, respectively). 

In the FEL discs, the EC50 value of T6P also occurred at a relatively low concentration of Suc (0.9488 mM), compared to Glc and G6P (1.958 and 1.760 mM Suc, respectively) (Figure 2F,G). This revealed that Suc had a high affinity for trehalose accumulation, and demonstrated that the difference between the specificity of Suc for T6P and Glc/G6P was greater in YL than in FEL discs (Figure 2F,G). 

The association constants (*K_a_* value) of Suc for Glc, T6P, and G6P were analyzed next (Figure 2D,E). In YL discs, Suc treatment showed no appreciable effect on G6P and Glc (*K_a_* = 0.17 × 10^−10^ mM^−1^ and *K_a_* = 0.19 × 10^−10^ mM^−1^, respectively); however, the effect of Suc on binding was strong (*K_a_* = 0.94 × 10^−9^ mM^−1^). A similar effect was observed in FEL discs, in which Suc bound more strongly to T6P (*K_a_* = 0.35 × 10^−10^ mM^−1^) than to Glc or G6P (*K_a_* = 0.166 × 10^−10^ and 0.143 × 10^−10^ mM^−1^, respectively). We therefore posited that Suc had a strong binding potential for T6P in the detached *N. tabacum* YL and FEL discs. 

### 2.5. Effects of Suc on the Sugar Signal Transduction Pathways Meditating Senescence

**Figure 3:**
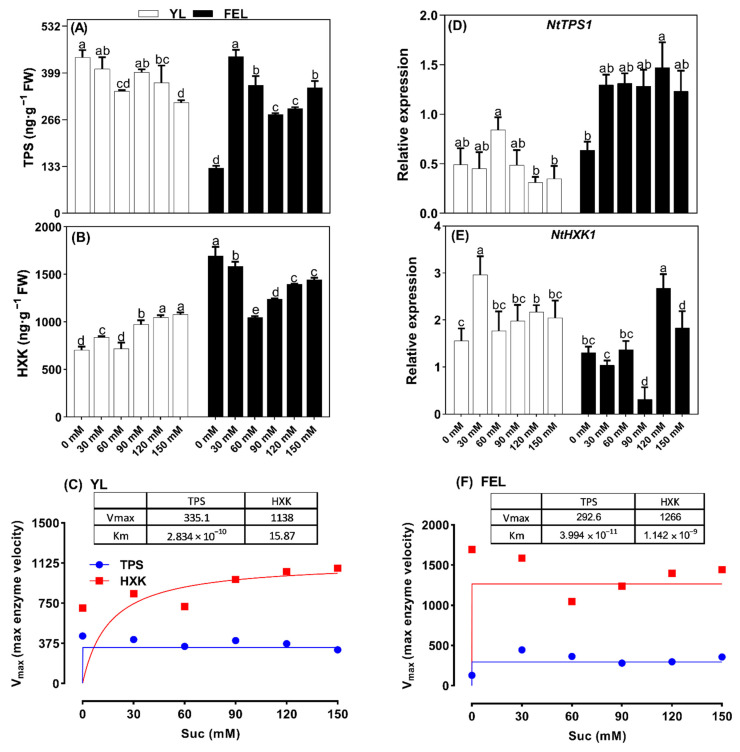
Dose-dependent effects of sucrose (Suc) on sugar metabolising enzymes. Trehalose 6-phosphate synthase (TPS) (**A**) and hexokinase (HXK) content (**B**) in detached young leaf (YL) and fully emerged leaf (FEL) discs of *N. tabacum* under a range of Suc concentrations. Relative expression of *NtTPS1* (**D**) and *NtHXK1* (**E**). Lowercase letters indicate statistically significant differences (*p* < 0.05). The Michaelis–Menten curves of TPS (**C**) and HXK (**F**) for Suc are shown in blue and red, respectively, in detached YL and FEL discs. The kinetic parameters of enzymes from YL and FEL discs are shown as inset tables.

Trehalose 6-phosphate synthase (TPS) and hexokinase (HXK) mRNA and enzyme abundance were measured to determine the physiological importance of senescence regulation through sugar signal transduction pathways (Figure 3A,E). In YL discs, TPS content significantly decreased by 21.7, 16.2, and 29.1% at 60, 120, and 150 mM Suc compared to untreated discs. Transcriptional abundance of *TPS1* showed a similar response at higher Suc levels; it was downregulated by 0.4- and 0.3-fold at 120 and 150 mM Suc, respectively. An exception was again observed at 60 mM Suc; in those samples, *TPS1* was upregulated (by 0.7-fold). This inconsistent response of *TPS1* may have been due to expression of TPS isogenes (Figure 3A). 

The effect of Suc on TPS content was different in FEL discs; compared to untreated discs, TPS levels first increased by 246.2 and 182.9% at 30 and 60 mM Suc, respectively, then decreased by 118.4, 131.3, and 177.2% at 90, 120, and 150 mM Suc, respectively (Figure 3A). In addition, transcriptional abundance of *TPS1* in the FEL was consistent with TPS enzyme content. *TPS1* was upregulated by 1.0-, 1.1-, 1.0-, 1.3-, and 0.9-fold at 30, 60, 90, 120, and 150 mM Suc, respectively. These results indicated that Suc affected TPS/*TPS1* signal transduction differently in YL and FEL discs. In YL discs, TPS decreased with increased Suc levels. However, in the FEL, TPS enzyme levels increased, which may be a different response mechanism associated with Glc-T6P signaling. Furthermore, the HXK content significantly increased by 19.0, 37.9, 48.2, and 48.3% in response to 30, 90, 120, and 150 mM Suc treatment, respectively. In addition, *HXK1* was significantly upregulated (0.9-fold change) at 30 mM Suc, and slightly upregulated (0.4-fold change) at 120 mM Suc. In FEL discs, HXK content was significantly reduced in response to Suc treatment; compared to untreated samples, HXK content decreased by 6.3, 38.2, 26.9, 17.5, and 14.8% at 30, 60, 90, 120, and 150 mM Suc, respectively. In contrast to HXK enzyme content, *HXK1* expression decreased by 0.8-fold at 90 mM Suc and increased by 1.0- and 0.4-fold at 120 and 150 mM Suc, respectively. The results clearly showed that the metabolic activity of HXK increased with low levels of Suc, but slightly decreased with higher Suc supply in the YL discs. *HXK1* transcript abundance did not have a strong or consistent response to Suc treatment in either YL or FEL discs. 

The differences in steady-state sensitivity of TPS and HXK revealed divergent behavior of Suc uptake potential in the detached YL and FEL discs. Several explanations for such behaviors (*V_max_* and *K_m_*) have previously been described [38]. A metabolic steady-state could be identified with an increased *V_max_* of TPS and HXK, and a decreased *K_m_* of Suc. *K_m_* is inversely related to *V_max_*, and the data showed that in YL discs, the *V_max_* of TPS (315.1 ng/g) was achieved with a lower *K_m_* for Suc (3 mM). HXK achieved a higher *V_max_* (1138 ng/g) with a high *K_m_* for Suc (15.87 mM). These data clearly showed that TPS responded quickly to Suc, but remained for only a short period of time after the saturation point. However, Suc affinity for HXK increased after the saturating concentration.

In FEL discs, TPS had a lower *V_max_* value (292.6 ng/g) at the lower *K_m_* for Suc (3 mM). However, HXK achieved the maximum velocity *V_max_* (1266 ng/g) at an even lower *K_m_* for Suc (1.14 mM), which clearly indicated that FEL was much more sensitive to HXK. This analysis showed that TPS content did not change much, and showed a linear trend after a saturation point of Suc in both the YL and FEL discs. However, the HXK velocity was altered more and increased in the YL; in the FEL, it did not increase after the saturation point. This demonstrated that hexose sugars accumulated as Suc concentrations increased in YL discs, accelerating the initiation of senescence. 

### 2.6. Involvement of T6P–SnRK1 Interactions in Regulation of Senescence in Detached Leaf Discs

**Figure 4:**
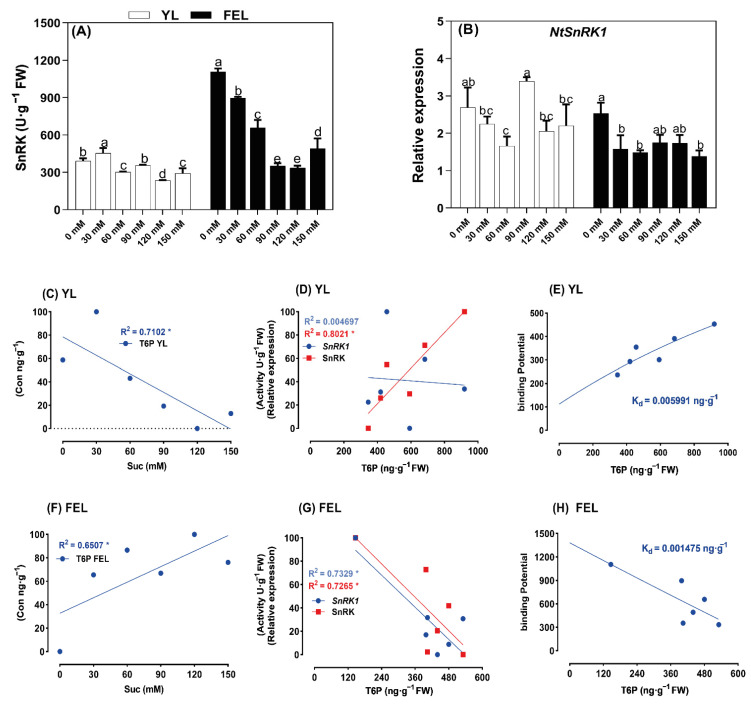
Exogenous sucrose (Suc)-dependent fluctuations in (**A**) SnRK enzyme activity and (**B**) *NtSnRK1* expression in detached *N. tabacum* young leaf (YL) and fully emerged leaf (FEL) discs. Lowercase letters represent statistically significant differences at *p* ≤ 0.05. Error bars indicate standard deviation. (**C**–**H**) Linear regression analyses: Suc and T6P in YL (**C**) and FEL discs (**F**); T6P and *NtSnRK1* (blue) or SnRK (red) in YL (**D**) and FEL discs (**G**); binding kinetics of the Suc-meditated T6P–SnRK complex in YL (**E**) and FEL discs (**H**). * *p*-value ≤ 0.005.

In current models of T6P involvement in plant development and metabolic control, T6P is strongly connected to Suc availability and inhibits *SnRK1*, which enhances the expression of biosynthetic genes [20]. Increases in T6P have been shown to accelerate leaf senescence, whereas decreases in T6P levels delay senescence [15], demonstrating the likely role of T6P in the induction of senescence in *N. tabacum* YL and FEL discs. Energy metabolism, SnRK activity, and *NtSnRK1* gene expression were therefore studied (Figure 4). In YL discs, Suc significantly affected SnRK activity and gene expression; SnRK activity was 15.9% higher at 30 mM Suc, but decreased by 23.0, 39.4, and 25.1% at 60, 120, and 150 mM Suc, respectively. No significant increase in SnRK activity was recorded at 90 mM Suc (Figure 4A). In addition, *NtSnRK1* was significantly upregulated (0.3-fold change) at 90 mM Suc, but significantly downregulated (0.4-fold change) at 60 mM Suc treatment. In contrast, no significant change was recorded at 30, 120, or 150 mM Suc treatments in detached YL discs (Figure 4B).

In FEL discs, SnRK activity and *SnRK1* gene expression both significantly decreased with increased Suc supply. SnRK decreased by 18.9, 55.5, 40.5, 68.0, and 69.7% at 30, 60, 90, 120, and 150 mM Suc, respectively (Figure 4A). Similarly, compared to 0 mM Suc, *NtSnRK1* was significantly downregulated (by 0.4-, 0.4-, and 0.5-fold) at 30, 60, and 150 mM Suc, respectively, but no significant changes were found at 90 and 120 mM Suc in the detached leaf discs (Figure 4A). 

To understand the interactions of T6P with SnRK, we performed a correlation analysis of Suc-T6P and SnRK in both leaf types. In YL discs, exogenous Suc was significantly negatively correlated with T6P (R^2^ = 0.7102, *p* < 0.05). However, in FEL discs, Suc was significantly positively correlated with T6P (R^2^ = 0.06507, *p* < 0.05) (Figure 4C,F). A linear regression analysis was performed to determine the inhibitory effect of T6P accumulation on SnRK activity and *NtSnRK1* gene expression. The results showed that in YL discs, SnRK activity was positively correlated with T6P (R^2^ = 0.8021, *p* < 0.05), but no significant correlation was observed with *NtSnRK1*. In contrast, in FEL discs, T6P was negatively correlated with both SnRK activity and *NtSnRK1* expression (R^2^ = 0.7265 and R^2^ = 0.7329, respectively; *p* < 0.05) (Figure 4D,G).

The correlation analysis showed negative and positive correlations of T6P and SnRK in detached YL and FEL discs. However, the *K_d_* of T6P and SnRK in senescent leaf discs was still unknown. Binding analyses showed that SnRK was inhibited at the dissociation constant of Suc in both YL discs (*K_d_* = 0.005991 mM; Figure 4E) and FEL discs (*K_d_* = 0.001475 mM; Figure 4H). These results showed that Suc induced SnRK accumulation in response to T6P in the YL discs. The *K_d_* value of T6P for inhibition of SnRK activity was lower in FEL than in YL discs. We therefore posited that levels of SnRK, which functions in energy homeostasis, slowly decreased in senescent FEL discs. However, SnRK was still stimulated by the Suc–T6P complex after reaching the threshold. 

## 3. Discussion 

Senescence is a complex process involving changes in cellular physiology, biochemistry, and gene expression [39]. Detached leaf discs are often used in senescence studies, even though senescence-induced changes in detached leaves may differ from those in intact leaves. Leaf discs represent a simple system to model assimilation of water and nutrients by an entire plant [40]; they are simple to handle and can be easily incubated under controlled conditions. For these reasons, detached tobacco leaves were used in this study [41]. 

Leaf senescence is the last stage of plant growth, and is affected by various genetic and environmental constraints [42]. Leaf yellowing is a visible symptom of senescence that is caused by loss of the green pigment chlorophyll (Chl) [42]. Chl fluorescence has been investigated to understand more about the functioning of the photosynthetic system, including the processes of light absorption, energy transformation, and senescence [43]. A previous study showed that the maximum quantum yield of PSII (F_v_/F_m_) was dramatically decreased during acclimation in *Alocasia amazonica* plantlets propagated with 6% instead of 3% Suc [44]. Similarly, we found that F_v_/F_m_ decreased in YL discs incubated in medium with a high Suc concentration (150 mM). Furthermore, F_v_/F_m_ also decreased in FEL discs at a low Suc concentration (60 mM). FEL discs are source leaves, which is why Suc caused an early reduction in F_v_/F_m_ (Figure 1B). The degradation of total Chl is positively related to F_v_/F_m_ [45]. Thus, prior to F_v_/F_m_ reduction, Chl markedly declined in response to high Suc content (120 and 150 mM) in YL discs, and in response to both low (60 mM) and high (120 mM) Suc content in FEL discs. The Chl decline was consistent with the decreased F_v_/F_m_ observed in both the YL and the FEL discs in response to Suc treatment. These results were supported by Li et al. [46], who demonstrated that senescence symptoms, such as a decline in F_v_/F_m_ and Chl, could be induced by Glc treatment. In addition, adding Suc to growth media was shown to prevent Chl accumulation in carrot callus culture [11]. 

The onset of senescence is frequently linked to increases in nutrient mobilization and other indicators such as biomass (fresh weight) [47]. The biomass of YL discs increased with 60 and 90 mM Suc treatments. Exogenous Suc also caused earlier biomass accumulation in YL discs as a result of increased Suc efflux, increasing sink strength and biomass accumulation [48]. This may cause leaf senescence by inducing excessive accumulation of Suc, hexose, and starch in *N. tabacum* leaves [49]. FEL discs also had higher biomass in response to 60 and 120 mM Suc treatment. This demonstrated that mass gains were comparatively slower in YL than in FEL discs. These findings indicated that exogenously applied Suc could induce senescence and promote both growth and dry-matter accumulation (Figure 1C). Senescence is the last stage of leaf development after a period of active photosynthesis and biomass production [50].

Progressive Chl loss and massive increases in EL and MDA content are general features of leaf senescence in plants [6,51]. In the present study, analysis of physiological parameters revealed that Suc application induced a marked decline in total Chl content. Subsequently, there was a rapid increase in EL at high Suc concentrations in YL discs and at a range of Suc concentrations in FEL discs. High concentrations of Suc also increased MDA content in detached YL and FEL discs (Figure 1D,E). It was previously reported that reduced Chl and levels of soluble sugar and Suc significantly increased accumulation of MDA [52]. 

As signaling molecules, sugars control the transcription of many plant genes [53]. *SAG12* was previously used as a molecular marker gene to study leaf senescence in plants [54]. Importantly, *SAG12*, which was previously thought to be sugar-inducible, was induced 900-fold by Glc [55]. In the present study, *NtSAG12* expression rapidly increased at low Suc levels, then decreased in YL discs with higher levels of exogenous Suc application (Figure 1F). It was also previously reported that Suc accumulation significantly downregulated *SAG12* [7]. Thus, *NtSAG12* suppression in YL discs may be associated with high hexose accumulation in response to Suc levels [23]. Moreover, *NtSAG12* expression was progressively induced by Suc supply in the FEL, which is considered ideal for promoting early induction of senescence. A decrease in photosynthesis, which is followed by SAG transcription, precedes leaf yellowing [7]. In this study, detached tobacco FEL discs showed high expression of *SAG12* in response to Suc application (Figure 1F). 

HXK acts as a sugar sensor in plants [56]; like Suc and Glc, it functions as a signal that modulates expression of hundreds of plant genes. In *Arabidopsis* leaves, Suc uptake stimulates the activity of enzymes involved in Suc synthesis and degradation [57]. Interestingly, Glc levels were increased in both YL and FEL discs. In YL discs, Suc treatment increased levels of Glc and G6P. However, T6P levels increased when exogenous Suc was low, then progressively declined. Changes in levels of G6P and T6P due to exogenous Suc application differed between YL and FEL discs, but they increased in both. It is possible that the observed altered sugar profiles were temporary, and were due to the rapid enzymatic regulation involved in leaf sugar metabolism [49]. Exogenous Suc is known to affect sugar metabolism [58]. The results of a previous study revealed that 10 mg/L exogenous Suc increased levels of soluble proteins, soluble sugar, fructose, glucose, and Suc in pea sprouts [59]. 

Dose-dependent glycolysis and phosphorylation curves showed that Suc had a lower EC50 for T6P than for G6P (Figure 2F,G). T6P levels in Suc-starved *Arabidopsis thaliana* seedlings in axenic culture were shown to increase from 18 to 482 pmol·g^−1^ fresh weight (FW) after Suc was added [60]. In the present study, the EC50 of T6P occurred at a relatively low concentration of Suc in YL discs. It was previously suggested that T6P acts as a signal of Suc availability, connecting plant growth and development to the metabolic status [37]. In FEL discs, Glc content increased at high Suc concentrations. Trehalose biosynthesis gradually increased in FEL discs after Suc absorption. Suc signaling is known to regulate plant metabolism and growth [61], although the Suc perception mechanism is uncertain [62]. A dose–response curve was generated to help resolve this (Figure 2D,E); it indicated that, compared to G6P, TPS phosphorylated T6P with a higher *K_a_* of Suc, showing that Suc application to FEL discs more effectively produced 50% of the maximal response of T6P. It was previously reported that trehalose mobilization and trehalase activation were efficiently triggered in a Gpr1-dependent manner by a low concentration of Suc (5 mM), but not Glc [31,63]; these analyses further supported the hypothesis that senescence is induced in FEL discs via Suc–T6P signaling pathways. In the TPS pathway, TPS converts G6P and UDP-glucose to T6P [64]. Our results therefore provided novel insights into signal transduction via Suc metabolism. 

TPS and HXK feedback responses during Suc metabolism in YL and FEL discs followed standard Michaelis–Menten enzyme kinetics (Figure 3C,F). The *V_max_* values of the two enzymes increased continuously with increasing Suc concentrations before reaching a constant value after a threshold concentration of Suc. The *V_max_* values of TPS were 335.1 ng/g in the YL and 2926 ng/g in the FEL; there were negligible differences in the associated *K_m_* values of Suc. In contrast, the high *V_max_* of HXK (1266 ng/g) was associated with the lowest *K_m_* of Suc (1.142 mM) in the FEL, compared to the *V_max_* value of 1138 ng/g at a *K_m_* of 15.87 mM of Suc in the YL (Figure 3C,F). These results confirmed that trehalose production via TPS-mediated G6P phosphorylation occurred more frequently in FEL than in YL discs. In addition, the velocity of HXK was highest at the lowest *K_m_* value of Suc in FEL discs. Thus, glycolysis and Glc phosphorylation were more robust in FEL discs compared to YL discs during induction of senescence by exogenous Suc application. As previously reported, the estimated *K_m_* of ShSUT1 for Suc was 2 mM. In sugarcane, ShSUT1 is expressed predominantly in mature leaves, which export Suc, and in the culm tissue, which actively synthesizes Suc [65]. Suc could theoretically trigger an increase in T6P levels by stimulating T6P synthesis via TPS [37]. *AtHXK1* is an accurate Glc sensor that mediates the effect of Glc (regardless of the phosphorylation status) through enzymatic activity [24]. This indicated that Suc activated the Glc-G6P-HXK pathway in YL, confirming that Suc-mediated induction of senescence in detached YL discs occurred via hexose accumulation. As previously reported, overexpression of HXK in *Arabidopsis* and tomato plants showed that it acted as a sugar sensor and accelerates leaf senescence. In contrast, transgenic *Arabidopsis* plants expressing antisense RNA targeting hexokinase transcripts showed delayed senescence [66]. Hexose accumulation was shown to coincide with a decline in Chl content, indicating that accelerated senescence was due to earlier hexose accumulation [20].

A comprehensive characterization previously explained the differential impacts of T6P, G1P, and G6P on *SnRK1* activity [67]. We applied this approach to Suc/T6P metabolism by connecting SnRK with TPS content based on *V_max_*, *K_a_*, and *K_d_*. This addressed the question of how two conserved central signaling mechanisms, Suc-T6P/SnRK1 and HXK, could be coordinated within central carbohydrate metabolism during induction of senescence in the YL and FEL discs. It has been reported that HXK activity represents a rate-limiting step in Suc cycling (i.e., the cleavage and resynthesis of Suc) [68], and TPS plays a role in trehalose metabolism and biosynthesis [16]. 

**Figure 5:**
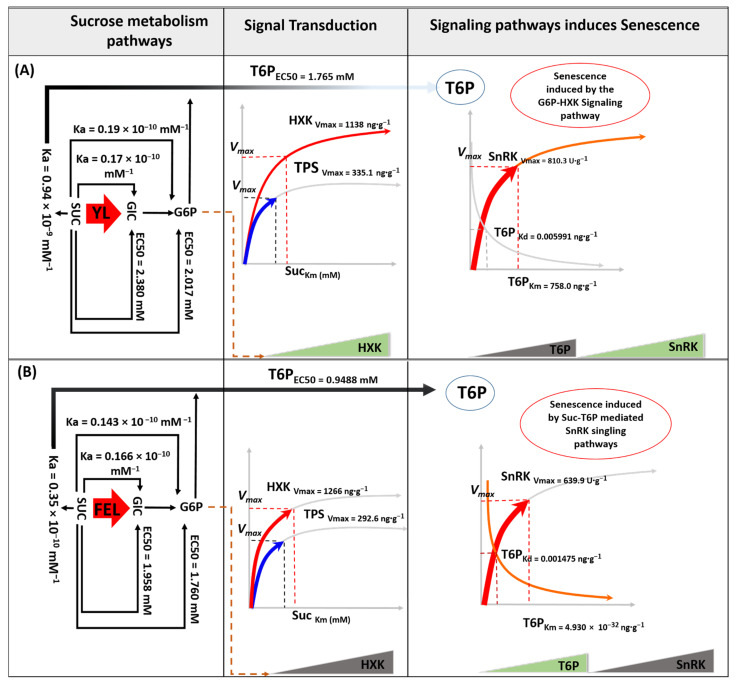
Schematic illustration of sugar metabolism and senescence induction in detached *N. tabacum* YL (**A**) and FEL (**B**) discs in response to exogenous sucrose (Suc) application. The schematic model represents simulated metabolite dynamics induced by external Suc application. This includes Suc breakdown and absorption by the detached leaf discs, metabolism via dynamic sugar signaling (specifically G6P-HXK and T6P-SnRK), and signaling pathway regulation in detached YL and FEL discs during induced senescence.

The enzyme kinetic analysis showed that in YL discs, trehalose responded at the lowest EC50 value of Suc (Figure 5). Compared to Glc and fructose, T6P levels increased ~40-fold in response to Suc supplementation, reaching levels much beyond those observed in nonstarved corban seedlings (control) [69]. Although the effectiveness of Suc-mediated T6P was higher (Figure 5A), the *V_max_* of TPS was linear after the *K_a_* of Suc reached a threshold. The findings of Thomas et al. (2014) supported these results; when T6P levels were between 0.3 and 0.5 nmol/g of FW and Suc levels were over 3.5 mol/g of FW, the relationship between these two metabolites was almost linear [38]. In the YL, after hydrolysis of Suc to Glc, HXK reached the maximum velocity with increasing Suc supply [70] (Figure 5A). HXK is a key enzyme in hexose metabolism [71]. It has a low *K_m_* for Glc, which is therefore phosphorylated as soon as it enters a cell. Phosphorylation of Glc by HXK is the first irreversible step in glycolysis, which is regulated only by excess G6P. If G6P accumulates in the cell, HXK is inhibited by feedback inhibition until the G6P is consumed [72]. This explains the observation in YL discs that hexose accumulation increased and T6P decreased with the rise in exogenous Suc supply. These data suggested that hexose accumulation is one of the factors leading to induction of senescence in detached YL discs. It has also been reported that high hexokinase activity accelerates senescence in tomato plants [73].

In FEL discs, the T6P was produced with a low half-maximal effective concentration EC50 value of Suc compared to Glc and Glc phosphates, and there was a higher Suc-induced T6P with a higher *K_a_* of Suc for TPS enzymes. T6P acts as a signal of Suc status in plants [74] and it helps to maintain Suc levels within an optimal range for the plant. Carbon-starved *A. thaliana* seedlings have very low levels of T6P (0.018 nmol/g of FW). However, T6P levels rose rapidly as Suc concentrations increased; within 15–30 min of exogenous Suc application, T6P content peaked at a level that was over 25 times higher than it was in C-starved seedlings [60]. Thus, we considered that T6P pathways may induce senescence in FEL discs. It was reported that T6P inhibited *Hxk1* and *Hxk2* expression, causing feedback inhibition of the influx of the Glc into glycolysis [75]. Moreover, T6P was found to inhibit the leaf senescence regulatory activity of *SnRK1*. Previously, increased T6P was shown to accelerate leaf senescence, whereas the opposite effect was observed when T6P levels were decreased [76]. In the present study, SnRK reached *V_max_* at the lowest predicted *K_m_* of the T6P substrate in FEL discs (Figure 5B, Supplementary Materials Figure S1). Moreover, SnRK enzymatic activity progressively decreased with lower *K_d_* values of T6P. Thus, by considering the reported metabolite concentrations and kinetics of T6P–SnRK1 and *HXK1* [38], we hypothesized that Suc-induced T6P accumulation may inhibit HXK and significantly inhibit SnRK activity in FEL discs; this could induce expression of *NtSAG12,* causing leaf senescence.

In summary, the kinetics of Suc metabolism in detached *N. tabacum* leaf discs confirmed that Suc directly affected senescence via induction of the T6P–SnRK complex in FEL discs; furthermore, Glc-G6P-HXK was assumed to be the form of hexose that accumulated in YL discs. 

## 4. Material and Methods 

### 4.1. Plant Material and Culture Conditions

The experiments were conducted with *Nicotiana tabacum* L. cv. “K326” seedlings at the five-leaf stage. Seedlings were obtained from the National Infrastructure for Crop Germplasm Resource Tobacco (Qingdao, China). The national serial number “00002266” was assigned to cultivar “K326” following a distinctness, uniformity, and stability (DUS) test. The experiments were conducted at the Tobacco Research Institute of the Chinese Academy of Agricultural Sciences (longitude 120°27′ E; latitude 36°8′ N). Characteristics of “K326” include late relative maturity, 40-inch plant height, a period of 64 days to flowering, and an average of 17.5 leaves per plant (Superior Tobacco Seeds; https://crosscreekseed.com, accessed on 3 February 2022). 

The detached leaf disc experiments were carried out as described by Zhao et al. (2018) [77]. The “K326” seedlings were grown in a greenhouse. Seeds were germinated in trays filled with a 1:1 combination of peat and vermiculite (*v*/*v*). Then, 20-day-old seedlings of comparable size were transplanted into 400 mL pots. Over the course of 11 days, 0.25× Hoagland nutritional solution was applied every three days. Leaf discs were floated in 0.5× Murashige and Skoog (MS) medium with a range of Suc concentrations [76]. The experiment was repeated twice; the preliminary experiment was performed with 0, 90, and 150 mM Suc with the pH of the MS medium adjusted to 5.6–5.8. Using data from the first experiment, the second experiment was conducted with 0, 30, 60, 90, 120, and 150 mM Suc; the pH of the medium was 5.6–5.7 before autoclaving. Seedlings with the most consistent morphology were chosen from each treatment group for further analyses. Detached YLs and FELs (the second and third leaves, respectively, from the top of the plant) were rinsed in tap water for 15 min, then transferred to an aseptic environment; leaves were next washed with 70% ethanol for 30–60 s, 3% NaClO for 15 min, and ddH_2_O two to three times. An 11 mm cork borer was used to punch out the leaf discs. Cultures were maintained in 50 mL Petri plates, each containing 50 *N. tabacum* YL and FEL discs and sealed with Parafilm. The photon flux density (PPFD) reaching the culture averaged 250 mol m^−2^ s^−1^, and the discs were cultured under a 16/8 h light/dark photoperiod. Plates holding the detached leaf discs were photographed on day 5 of incubation, and samples for downstream analyses were all collected on the same day. Leaf samples were quickly washed with water, then frozen in liquid nitrogen for analysis of their physiology, biochemistry, and gene expression.

### 4.2. Chlorophyll Quantification

All leaves were sampled at the same time. There were three biological repeats of the experiment to minimize experimental error. Each biological repeat comprised three leaf discs from a single Petri plate as a composite sample. Chl content was determined as described by Zhao et al. (2018) and Papista et al. (2002) [77,78]. The weight of each leaf disc was precisely measured, then leaf discs were entirely immersed in 10 mL absolute ethanol in 10 mL centrifuge tubes. Tubes were incubated in the dark at ambient temperature for 24–48 h. A blank solution was used as the control. Absorbance was measured at 665 and 649 nm in each sample using an Infinite M200PRO spectrophotometer (TECAN). The following formula was used to determine the Chl content of the *N. tabacum* leaf discs:

C (mg/g) = (Ca + Cb) × (V/W × g)

where Ca = 13.95 × X1 − 6.88Y × Y1; Cb = 24.96 × Y1−7.32 × X1; V = 10 mL absolute ethanol; Ca = Chl a; Cb = Chl b; and W is the fresh mass of the leaves. X1 and Y1 are the absorbance values at 665 and 649 nm, respectively [77]. 

### 4.3. Determination of Electrolyte Leakage and Malondialdehyde (MDA) Content

Electrolyte leakage was determined as previously described [77]. Centrifuge tubes (50 mL capacity) were filled with 40 mL deionized water, and three detached leaf discs (representing one sample) were washed, dried, then placed into the centrifuge tubes. There were three biological replicates for each sample. Tubes containing samples were incubated for 30 min in a water bath at 25 °C. The initial electrical conductivity (EC_initial_) of each sample was measured and recorded (GMH 3460 Conductivity Meter, OHAUS). The centrifuge tubes were then immersed in a water bath at 96 °C. After incubating the leaf discs for 15 min, the tubes were cooled to 25 °C, after which the EC_final_ was measured. Relative electrical conductivity was recorded as the percentage of the first electrical conductivity value (EC_initial_) over the final value (EC_final_):

% relative EC = (EC_initial_/EC_final_) × 100

MDA levels were determined using a previously described technique [79]. Briefly, 1.0 g leaf discs were crushed on ice in 10 mL of a 10% trichloroacetic acid solution. Samples were centrifuged at 5000× *g,* and 1 mL of the supernatant was retained. Thiobarbituric acid solution (3 mL, 0.6%) was then added to each sample, and samples were heated at 95 °C for 15 min in a water bath. After rapid cooling, the absorbance of each sample was measured at 450, 532, and 600 nm. MDA levels were calculated using the following formula [80]:

*_C_* (μmol/L) = 6.45 × (OD_532_ − OD_600_) − 0.56 × OD_450_

### 4.4. Chlorophyll Fluorescence Measurements

Chl fluorescence was analyzed using a pulse-modulated imaging fluorometer (FluorCam 700MF; Photon Systems Instruments, Brno, Czech Republic). Minimum fluoresce values (F_0_) were recorded by exposing the plants to modulated red light before a saturating flash of white light for a duration of 0.8 s. These data were used to capture maximum fluorescence (F_m_) and to calculate maximum photosynthetic efficiency (F_v_/F_m_) of the leaf discs [80]. 

### 4.5. Sugar Metabolites and Enzyme Quantification Analysis 

ELISA Kits from Nanjing Camilo Bioengineering Co., Ltd. (Nanjing, China). (www.njkmlbio.com, accessed on 11 January 2022) were used for analysis of sugar contents. Phosphate-buffered saline (PBS) at 0.01 M was prepared by mixing two saline solutions (0.2 M Na_2_HPO_4_·12H_2_O and 0.2 M NaH_2_PO_4_·12H_2_O), and the pH was adjusted to 7.4. PBS (2 mL of 0.01 M) was added at a proportion of 5–10 mL per gram of fresh tissue weight, homogenized using a tissue lyser, then mixed on ice. Samples were centrifuged for 10 min at 5000–10,000 rpm. The prepared samples and the standard reactions (for each substrate–enzyme pair) were added to 96-well ELISA plates precoated with appropriate antibodies and incubated at 37 °C for 90 min. Plates were then washed twice with washing buffer (1:25), the 100 μL biotinylated antibody solution (1:100) specific to each substrate–enzyme pair was added, and plates were incubated at 37 °C for 60 min. Plates were then washed three times with washing buffer. Next, 100 μL of the enzyme conjugate solution was added to each well (except the blank/control well) before incubation at 37 °C for 30 min. Plates were washed five times before 100 μL of color reagent was added, then incubated at 37 °C for 30 min. The color differences between the standards and the samples were then observed. When a color gradient was observed for the standard solution reactions, stopping solution was added; this occurred within 30 min. Enzymes and substrates were then added for each reaction: HXK (Cat No. 2Pl-KMLJ92052p), TPS (Cat No. 2Pl-KMLJ92095p), or SNRK (Cat No. 2Pl-KMLJ92051p), and Glc (Cat No. 2Pl-KMLJ91833p), G6P (Cat No. 2Pl-KMLJ91348p), or T6P (Cat No. 2Pl-KMLJ92068p). Absorbance values were quantified at 450 nm with a microplate reader (Spectrophotometer, 1510-03148C, Thermo Fisher Scientific, Vantaa, Finland). 

The chemical compositions of the reagents used in these experiments are listed in Supplementary Materials Online Resource File S1 (reagents list). 

### 4.6. Mathematical Modeling and Data Analysis 

Mathematical enzyme kinetic analyses were performed using GraphPad Prism version 8.0.2 (GraphPad Software, San Diego, CA, USA). 

**Dose–Response Curves**. EC50 is the effective concentration of a compound that induces half of the maximum enzyme activity; i.e., the concentration of agonist that induces a response halfway between the baseline and maximum responses [81]. Dose–response curves were fitted with the Hill Equation:

E/E_max_ = 1/1 + (EC_50_/[A])^n^

where E is the magnitude of the response, [A] is the drug concentration (or stimulus intensity), EC50 is the drug concentration that produces a response that is 50% of the maximal response, and n is the Hill coefficient [82].

**Michaelis–Menten saturation curve**. Enzyme reaction kinetics are measured with the Michaelis constant, which is equal to the substrate concentration at which the reaction occurs at half of the maximum rate. A Michaelis–Menten saturation curve demonstrates the relationship between substrate concentration and enzyme reaction rate. The Michaelis–Menten equation is as follows:

*V* = *V_max_*[*S*]/*K_M_* + [*S*]

where *V_max_* is the peak velocity at the maximum (i.e., saturating) substrate concentration, *K_M_* is the substrate concentration at which the response velocity is 50% of *V_max_* (the Michaelis constant (frequently abbreviated as *K_S_*), and S is the substrate [83].

**Binding affinity kinetics.** We used the equation in GraphPad Prism version 8.0.2 called “Association kinetics-One conc. of hot”:

*K_d_ = K_off_/K_on_*

where *K_a_* or *K_on_* is the association rate constant in M^−1^ min^−1^ and *K_d_* is the equilibrium dissociation constant in M, computed as *K_off_*/*K_on_.*

*K_d_* (the dissociation constant) is the ligand concentration at which half of the ligand-binding sites on the protein are occupied in the system equilibrium. It is calculated by dividing the *K_off_* value by the *K_on_* value. The units for *K_d_* are measured in M; smaller *K_d_* values indicate a greater affinity between proteins. *K_a_* is the association constant (the inverse of *K_d_*) and is calculated by dividing *K_on_* by *K_off_* [84].

### 4.7. Transcription of Senescence-Associated, Sugar Sensor, and Sugar Metabolism Genes

Total RNA was extracted from three biological replicates of YL and FEL discs in each Suc treatment group using the MiniBEST Plant RNA Extraction Kit (TaKaRa, Japan). RNA concentration was measured with a Nanophotometer P330 (Implen, Munich, Germany), and RNA quality was visualized with agarose gel electrophoresis. Using 1 µg aliquots of RNA, all samples were diluted to 200 ng; 1 µL of each RNA sample was then used in quantitative reverse transcription (qRT)-PCR analysis with one-step SYBR Green Real-time PCR Master Mix Reagent Kit (Vazyme, Cat no. Q221-01, Nanjing, China) and the BioRed CFX96^TM^ system (BIO-RAD, Hercules, CA, USA) following the manufacturer’s instructions. The PCR program was as follows: reverse transcription, 50 cycles of 50 °C for 3 min; pre-denaturation, 95 °C for 30 s; PCR, 40 cycles of 95 °C for 10 s and 60 °C for 30 s; melting curve, 95 °C for 15 s. In addition, a melting-curve protocol from 60 °C to 95 °C was performed to detect the single gene-specific peak for all tested primer pairs. Gene-specific primers (Supplementary Table S1) were designed using Primer-BLAST (http:// www.ncbi.nlm.nih.gov/tools/primer-blast), accessed on 10 November 2021).

The relative expression levels of *NtCP1/SAG12* (NM_001325277.1), *NtHXK1* (NM_001325809.1), *NtSnRK1* (XM_016583314.1), and *TPS1* (XM_016635437.1) were calculated using the 2^−∆∆CT^ method [85] with actin serving as the internal control. The averages and standard deviation values for the target genes were calculated from three biological and three technical replicates each. 

### 4.8. Data Representation and Statistical Analysis

The data were evaluated using a two-way analysis of variance (ANOVA). The Chl content, F_v_/F_m_, MDA content, electrolyte leakage, and gene expression for *N. tabacum* leaf discs were compared between control and test groups with a Student’s *t*-test (Statistix 8.0, Analytical Software, Tallahassee, FL, USA). A *p*-value of 0.05 was considered statistically significant. The mathematical kinetic analysis was performed using Graph Pad Prism version 8.0.2 (GraphPad Software, San Diego, CA, USA). 

## 5. Conclusions

Suc sensing and signaling related to sugar precursor levels in detached *N. tabacum* leaf discs varied between YL and FEL samples. From sugar profile alterations and mathematical kinetic analysis of the metabolites, we determined that Suc caused accumulation of T6P in FEL discs and steadily increased hexokinase velocity in YL discs. Kinetic analysis also confirmed that Suc induced T6P accumulation with high-affinity *K_d_* values in YL (0.005991 mM) and FEL discs (0.001475 mM). However, compared to FEL, the key Glc component, hexose, was progressively accumulated (*V_max_ =* 1138 ng^.^g^−1^ FW) in the YL discs, leading to the hypothesis that the hexose-dependent signaling pathway accelerated senescence. Additionally, traits related to senescence (Chl, F_v_/F_m_, MDA, and EL) changed in response to concentrations of Suc ≥ 90 mM in the YL. In the FEL, F_v_/F_m_, Chl, and EL were altered at concentrations of Suc < 90 mM, whereas MDA and *NtSAG12* expression were altered at concentrations ≥90 mM. Thus, 90 mM of Suc was determined to be the optimal concentration to initiate senescence in the YL, which was considered to be sink tissue. Moreover, 60 mM Suc was the best concentration for initiating senescence in the FEL, which was considered a source tissue. This research lays a solid foundation for further studies to fully explain the dose-dependent trehalose and hexose signaling pathways and their underlying mechanisms in regulating leaf senescence in *N. tabacum.*

## Data Availability

The data presented in this study are available on request from the corresponding author.

## Supplementary Materials

The following are available online at https://www.mdpi.com/article/10.3390/ijms23126498/s1.
